# Translating Pharmacokinetics and Pharmacodynamics into Clinical Decision-Making: A Practical Guide to the Use of Risperidone ISM^®^ in Schizophrenia

**DOI:** 10.3390/jcm15166393

**Published:** 2026-08-18

**Authors:** Marco Solmi, Olivier Corbeil, Maïa Miguelez, Ric M. Procyshyn, Pierre Blier, Maxime Barakat, Christoph U. Correll

**Affiliations:** 1 SCIENCES Lab, Department of Psychiatry, Faculty of Medicine, University of Ottawa, Ottawa, ON K1H 8M5, Canada; olivier.corbeil.1@ulaval.ca; 2 Department of Mental Health, The Ottawa Hospital, Ottawa, ON K1H 8L6, Canada; 3 Ottawa Hospital Research Institute (OHRI), Ottawa, ON K1H 8L6, Canada; 4 Department of Child and Adolescent Psychiatry, Psychosomatics and Psychotherapy, Charité Universitätsmedizin—Berlin, 10117 Berlin, Germany; 5 Department of Pharmacy, Institut Universitaire en Santé Mentale de Québec, Quebec, QC G1J 2G3, Canada; 6 Faculty of Pharmacy, Université Laval, Quebec, QC G1V 0A6, Canada; 7 Bausch Health, Canada, Inc., Laval, QC H7L 4A8, Canada; 8 Department of Psychiatry, University of British Columbia, Vancouver, BC V6T 2A1, Canada; 9 The Royal Ottawa Mental Health Care Center, Ottawa, ON K1Z 7K4, Canada; 10 Department of Psychiatry, McGill University, Montreal, QC H3A 1A1, Canada; 11 Department of Psychiatry Research, The Zucker Hillside Hospital, New York, NY 11004, USA; 12 Department of Psychiatry and Molecular Medicine, Donald and Barbara Zucker School of Medicine at Hofstra/Northwell, Hempstead, NY 11549, USA; 13 German Center for Mental Health (DZPG), Partner Site Berlin, 10117 Berlin, Germany; 14 Einstein Center for Youth Mental Health (ECYM), 10117 Berlin, Germany

**Keywords:** schizophrenia, risperidone ISM, long-acting injectable antipsychotics, pharmacokinetics, pharmacodynamics, antipsychotic switching, acute exacerbation, efficacy, safety, tolerability, adherence, dopamine D2 receptor occupancy

## Abstract

Nonadherence to medications for psychosis is a major challenge in the management of schizophrenia and is associated with an increased risk of relapse, hospitalization, functional deterioration, and premature death. Although long-acting injectable (LAI) medications for psychosis improve treatment continuity, important differences exist among LAIs regarding initiation regimen, attainment of therapeutic blood level exposure, and steady state, dosing, and switching. Risperidone ISM^®^ is an intramuscular LAI administered every four weeks, designed to provide rapid and sustained therapeutic exposure without the need for loading doses or oral supplementation. This narrative review translates the pharmacokinetic (PK) and pharmacodynamic (PD) profile of risperidone ISM^®^ into expert-informed guidance for treatment initiation, dose optimization, switching, and ongoing schizophrenia management. Risperidone ISM^®^ achieves therapeutic exposure within hours of administration and maintains plasma concentrations and dopamine D2 receptor occupancy at levels associated with antipsychotic efficacy throughout the dosing interval, supporting continuous antipsychotic coverage and stability. Its PK profile, as characterized in clinical studies, and simplified initiation regimen may facilitate clinical decision-making across treatment settings, including transitions from oral risperidone or other medications for psychosis and continuity of care following hospitalization or relapse, while supporting symptom stability, functioning, and patient engagement. By translating PK/PD characteristics into practical clinical implications, this review aims to support clinicians in optimizing the use of risperidone ISM^®^ as part of individualized, recovery-oriented care in the short- and long-term management of people living with schizophrenia across settings. The recommendations presented reflect expert interpretation of the available PK/PD, clinical trial, observational, and product-label evidence, supplemented by clinical experience.

## 1. Introduction

Schizophrenia is a chronic and disabling condition affecting approximately 1% of the global population [1,2]. Individuals living with schizophrenia often experience recurring relapses, with up to 82% experiencing relapse within 5 years of diagnosis, and rates being similarly high in subsequent years [3]. Relapse in schizophrenia may have deleterious neurobiological impacts and contribute to the development of treatment resistance [2], as well as premature mortality [4]. Relapses can also severely disrupt patients’ lives through worsening symptoms, loss of functional gains, self-harm, and hospitalization, for example [3], creating a large clinical and economic burden on healthcare systems [3,5,6].

Maintaining continuous treatment can be challenging for many people living with chronic illnesses; indeed, 50% of patients do not take their medication as prescribed after 6 months [7]. For people with psychotic disorders, additional illness- and treatment-related challenges may affect treatment continuity, including limited insight, negative symptoms, substance use, adverse effects from medication, and practical barriers to care [7]. In these patients, nonadherence is associated with an increased risk of worsening symptoms, relapse, and even premature mortality [8,9,10].

Long-acting injectable (LAI) formulations of medications for psychosis have been developed to support treatment adherence, continuity, and, ultimately, effectiveness (Table 1) [2,8]. Indeed, LAI use has been shown to reduce relapse rates, hospitalizations, emergency department visits, and mortality [8,9,10,11]. LAIs may also play an important recovery-oriented role by helping patients maintain symptom stability, social participation, psychosocial functioning, quality of life, and treatment engagement over time [12,13,14,15,16,17]. Additionally, earlier use of LAIs has been suggested as having a particularly favorable impact on the illness trajectory [2,18,19,20]. However, the majority of LAIs are characterized by a delayed onset of action, and many require a loading dose or oral supplementation upon initiation to achieve therapeutic concentrations [2,21]. In addition, potential logistical and operational challenges for some LAIs may limit their use, including storage requirements (such as refrigeration), complex reconstitution techniques, limitations on injection site, and coordination of care for the patient [20,22]. Other barriers to LAI use include patient concerns about needles, treatment costs, and under-representation in clinical practice guidelines, despite robust efficacy and safety data from clinical and real-world trials [19,20,22].

First-generation LAIs (e.g., fluphenazine decanoate and haloperidol decanoate) are supplied in an oil-based formulation, which can be painful at the injection site [26]. Second-generation LAIs are mostly water-based, with some requiring oral supplementation during the first few weeks of treatment or a loading dose strategy (Table 1). For example, risperidone biweekly LAI requires oral supplementation during the first 3 weeks, whereas paliperidone monthly LAI uses a two-dose loading dose injection regimen without oral supplementation. Aripiprazole monthly LAI 400 mg requires either a 14-day oral supplementation after the first injection, or can be initiated using a two-injection initiation strategy [26,27,28]. Additionally, although not available in Canada, olanzapine pamoate requires a 3-h post-injection observation period because of the risk of post-injection delirium/sedation syndrome, limiting its utility and increasing resource burden [26,29]. LAI formulations without oral supplementation or loading doses may therefore offer advantages over existing formulations, by making initiation simpler for patients and clinicians and by increasing the speed at which therapeutic levels in the bloodstream are reached [30]. This may be of particular interest in settings where timing of treatment initiation is a key factor.

Risperidone ISM^®^ is an LAI based on an in situ matrix drug-delivery system that produces an initial rapid release followed by a slower sustained release without requiring oral supplementation or a loading dose upon initiation [2,30]. The pharmacokinetic (PK) and pharmacodynamic (PD) profile of risperidone ISM^®^ has been evaluated in several clinical and modeling studies, together with clinical trial and observational evidence regarding its efficacy and tolerability [27]. Nevertheless, clinicians may be less familiar with the practical implications of this formulation and its PK/PD profile. Although risperidone ISM^®^ was approved in Europe in 2022 [27], clinical experience in North America remains limited because it is not currently marketed in the United States and became available in Canada only recently [27]. Direct head-to-head evidence comparing risperidone ISM^®^ with other LAIs is also limited, and comparative findings derived from indirect analyses, modeling studies, or evidence concerning other risperidone formulations should therefore be interpreted cautiously.

In this article, we summarize existing challenges in schizophrenia treatment and translate the available evidence on the PK/PD profile of the risperidone ISM^®^ into expert-informed practical guidance on initiation, dosing, switching, and ongoing management. The comparison with other LAIs is intended to provide clinical context rather than a comprehensive comparative review. The recommendations reflect expert interpretation of the available PK/PD, clinical trial, observational, and product-label evidence, supplemented by clinical experience, and should not be interpreted as formal evidence-based treatment guidelines. The overall goal of the article is to support individualized decision-making, treatment continuity, symptom stability, and patient-centered care.

## 2. Overview of Risperidone ISM^®^

Risperidone is a psychotropic benzisoxazole derivative with a strong antagonistic affinity for dopamine D2, serotonin 5-HT2, and NAα-2 receptors, and a half-life of 3 h [1,30]. The pharmacological effects of risperidone also depend on its primary active metabolite 9-hydroxyrisperidone (9-OH-RIS, also known as paliperidone), which has a longer terminal half-life (20 h), higher affinity for D2 receptors, and reduced affinity for α1-adrenoceptor and 5-HT2-receptors compared with risperidone itself [30]. In risperidone PK studies, the term “active moiety” is used to refer to the sum of risperidone and 9-OH-RIS, as both are active and clinically relevant [30].

**Figure 1:**
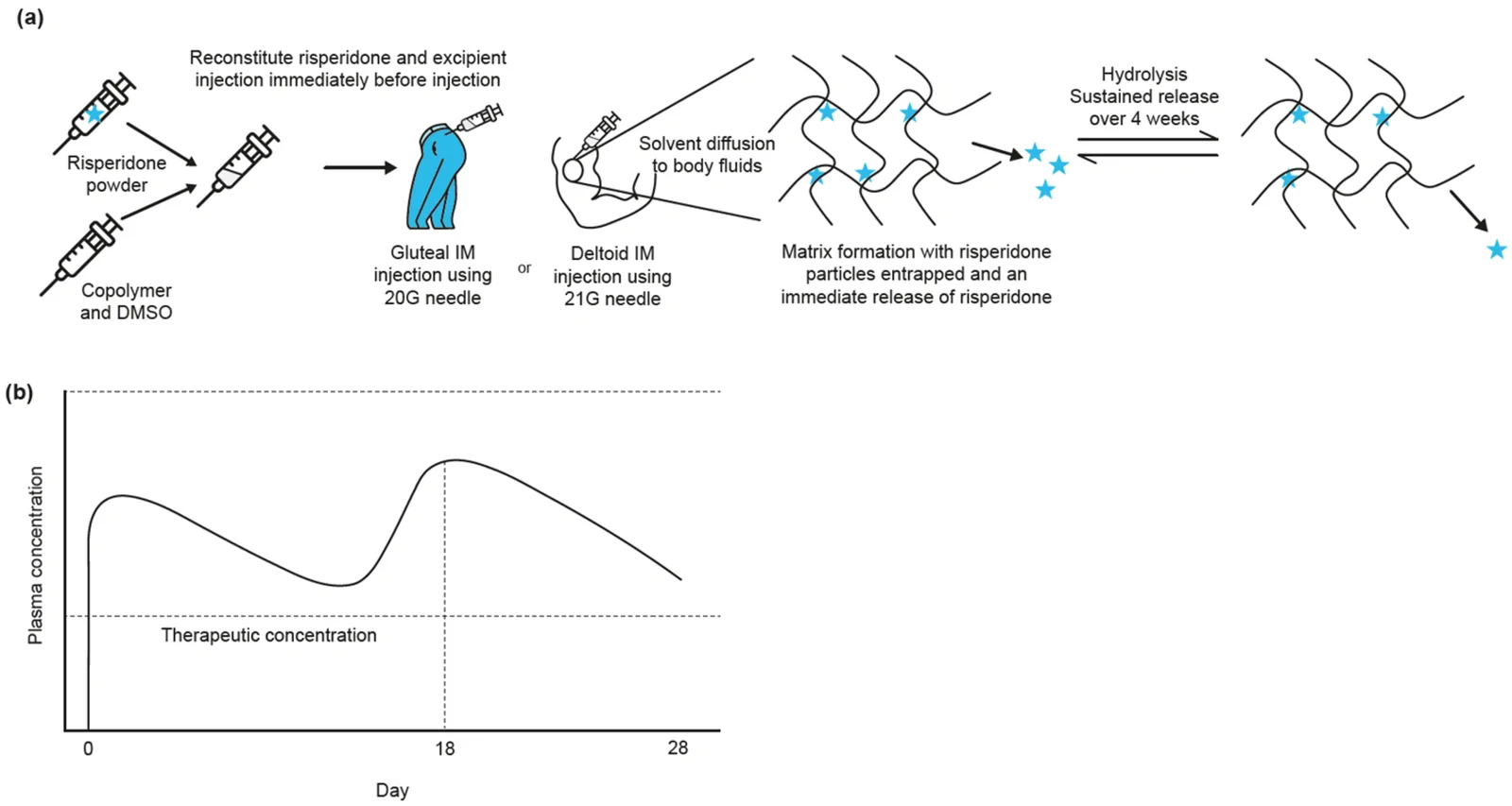
Schematic representation of the risperidone ISM^®^ formulation and its biphasic release profile [1,27,30]. (**a**) Following reconstitution and IM administration, risperidone ISM^®^ undergoes solvent exchange, resulting in polymer precipitation and formation of a biodegradable in situ matrix which entraps risperidone (blue stars), resulting in an immediate rapid release of the active moiety. Matrix biodegradation then enables further controlled risperidone release over the 4-week dosing interval. The resulting biphasic release pattern comprises an initial rapid release phase, supporting early therapeutic exposure, followed by sustained release from the polymeric matrix throughout the dosing period. (**b**) Schematic of risperidone ISM biphasic release pattern. The horizontal dashed lines indicate the therapeutic window, and the vertical dashed line indicates the second peak concentration [33]. DMSO; dimethyl sulfoxide; IM, intramuscular.

The first risperidone LAI formulation consisted of risperidone microspheres, which require injections every 2 weeks and 3 weeks of oral supplementation upon initiation. Since then, other risperidone LAI formulations have been approved, extending the dosing interval to up to 1 month [30]. Risperidone ISM^®^ is a LAI administered every 4 weeks that utilizes ISM^®^, a novel technology developed by Laboratorios Farmacéuticos Rovi (Spain). This technology is based on the formation of a biodegradable and stable polymeric matrix system that entraps microparticles of the active ingredient in situ, providing an initial rapid release of active moiety that reaches therapeutic plasma concentrations within 2 h [31], followed by a sustained release throughout the dosing period (Figure 1) [1]. Historically, the acronym ISM^®^ was defined as “in situ microparticles”, or in earlier reports “in situ microimplants”, to represent the microparticles created inside the body following injection [1,32]. However, the term “in situ matrix” may more accurately reflect the underlying drug-delivery technology and help distinguish risperidone ISM^®^ from other LAIs, particularly risperidone microspheres.

The risperidone ISM^®^ formulation is supplied as a monthly (28-day) two-syringe kit for administration by intramuscular (IM) injection in either the gluteal or deltoid muscle [27]. The formulation must first be reconstituted into an injectable suspension containing the active ingredient along with two excipients—a biocompatible poly(lactic-co-glycolic acid) copolymer and dimethyl sulfoxide (DMSO) as a solvent.

Following administration, a bidirectional solvent exchange process is initiated [1,30]. DMSO, together with a small fraction of dissolved risperidone, diffuses from the injection depot into the surrounding tissue. Concurrently, water from the extracellular fluid diffuses into the formulation, promoting rapid polymer precipitation and formation of a solid polymeric matrix in which risperidone is entrapped. Subsequent gradual biodegradation of this matrix, primarily through hydrolysis, enables sustained, controlled release of risperidone over time (Figure 1). Overall, this solvent exchange process gives rise to a biphasic release profile of the active moiety [30,34]. The initial release phase is associated with a first plasma concentration peak approximately 24–48 h after administration, whereas the later release phase is associated with a second peak between Days 18 and 25 [32,33,35]. Thereafter, plasma concentrations decline gradually over time [32].

Owing to its relatively short apparent elimination half-life (~8–11 days), risperidone ISM^®^ achieves plasma concentrations after the first injection that are comparable to oral risperidone steady-state exposure, with steady-state concentrations achieved from the first dosing cycle [32]. This contrasts with some other LAI formulations, which require several dosing cycles before steady-state concentrations are achieved [2,21]. Importantly, plasma concentrations remain within therapeutic levels throughout the dosing interval, with exposure consistent across both gluteal and deltoid injection sites [31,32].

For most D2 antagonist medications for psychosis, D2 receptor occupancy in the range of 65 to 80% is generally considered to optimize antipsychotic efficacy while minimizing the risk of extrapyramidal symptoms [35,36]. Timely delivery of LAIs, including risperidone formulations, is important to achieve and maintain this target and support a sustained antipsychotic effect [35]. For risperidone, this therapeutic occupancy range has been associated with active moiety plasma concentrations of approximately 20–60 ng/mL, although a lower threshold of >7.5 ng/mL has also been used to define therapeutically relevant exposure in risperidone ISM^®^ studies [32,37]. Risperidone ISM^®^ has been shown to reach this occupancy threshold during its early diffusion phase, within approximately 2 h of administration, thereby eliminating the need for oral supplementation or a loading dose. In addition, it maintains D2 receptor occupancy above 65% throughout the dosing interval [31,35].

### 2.1. Efficacy of Risperidone ISM^®^

Risperidone ISM^®^ has demonstrated efficacy in managing the core symptoms of schizophrenia [38,39]. In the multicenter, placebo-controlled PRISMA-3 trial, patients with an acute exacerbation of schizophrenia, there was a statistically significant difference of both doses (75 mg and 100 mg) of risperidone ISM^®^ versus placebo on the Positive and Negative Syndrome Scale (PANSS) total score mean change from baseline to Day 85, with placebo-adjust differences of −13.0 (*p* < 0.0001) for the 75 mg dose and of −13.3 (*p* < 0.0001) for the 100 mg dose [38]. Statistically significant improvements in PANSS total score mean change against placebo, as well as overall response rate, were seen as early as Day 8 for risperidone ISM^®^ 100 mg (LS Mean difference, 95% CI: −3.9, −6.4 to −1.5; *p* = 0.001) and by Day 15 for risperidone ISM^®^ 75 mg, which then remained significant until the end of the study (Day 85) [38]. By Day 85, overall response rates were significantly greater with risperidone ISM^®^ (75 mg dose group: 39.2%, 100 mg dose group: 33.8%, *p* < 0.0001), translating into a number-needed-to-treat of three for both doses.

These improvements in symptomatology were also observed in the PRISMA-3 open-label extension, where improvements in mean PANSS score were observed at Day 365 compared with baseline, and were comparable to baseline mean values even for patients clinically stable on oral risperidone at the start of the study [39]. In the RESHAPE study—a European real-world, multicenter study of adults diagnosed with schizophrenia—patients who were hospitalized after an acute exacerbation and then treated with risperidone ISM^®^ were observed to show rapid and sustained symptom improvement, with reported clinically meaningful reductions in psychotic symptom severity scores as early as Day 8 and continued improvement through Day 56 [40]. Because RESHAPE was a prospective, noninterventional study without a randomized comparator, its findings should be interpreted as observational associations [40]. Residual confounding and selection effects cannot be excluded; however, the study provides evidence from a patient population and treatment setting that may be more representative of routine clinical practical than randomized clinical trials. The RESHAPE study was also not a formal post-marketing surveillance study. Dedicated post-marketing pharmacovigilance data for risperidone ISM^®^ remain limited.

In addition to these efficacy measures, risperidone ISM^®^ improved personal and social functioning versus placebo in the PRISMA-3 trial, with significant improvements across multiple Personal and Social Performance (PSP) scale domains from as early as Day 29, and across various aspects of patient-reported well-being, particularly social integration and mental functioning [41]. These improvements were maintained or further enhanced during the 12-month open-label extension, with sustained improvements in PSP domains and well-being [41]. In a real-world setting, treatment with risperidone ISM was also associated with improvements in personal and social functioning, high patient satisfaction and improved therapeutic alliance, as demonstrated by the RESHAPE trial [40].

Together, these data suggest that risperidone ISM^®^ could form an effective therapeutic strategy in providing symptomatic improvements for patients with schizophrenia [38,39,40]. Risperidone ISM efficacy data are in line with evidence on oral risperidone, which has been shown to be superior to placebo (and to several other oral medications for psychosis) in improving symptoms in those with acute schizophrenia [42].

### 2.2. Safety and Tolerability of Risperidone ISM^®^

As with any pharmacological intervention, adverse events associated with medications for psychosis must be considered alongside efficacy before treatment commences, especially as these drugs need to be taken indefinitely [43]. Different medications for psychosis are associated with different side-effect profiles depending on their mechanism of action and receptor-binding profiles (Table 1 and Table 2) [43]. Common side effects with selected medications for psychosis are described in Table 2. Understanding the safety and tolerability profile of risperidone ISM^®^ is essential for evaluating its role in schizophrenia management.

Across the PRISMA clinical trials, treatment-related adverse events (TRAEs) were common, but generally mild to moderate [31,33,38,39]. Injection site pain was the most frequently reported TRAE in both the Phase 1 and 2 trials (PRISMA-1 and -2, respectively); oromandibular dystonia and somnolence/sedation were additional frequently reported TRAEs in the Phase 2 trial [31,33]. In the Phase 3 trial (PRISMA-3), the most frequently reported TRAEs were increased blood prolactin, hyperprolactinemia, akathisia, and headache [38]. This TRAE pattern was broadly consistent in the 1-year open-label extension of PRISMA-3, although additional commonly reported TRAEs included asthenia, weight gain, and insomnia [39].

Additionally, the RESHAPE study reported a safety and tolerability profile considered favorable, with 16.7% of patients reporting TRAEs, most of which were mild and most frequently consisted of weight gain and injection-site pain. The observed TRAEs were consistent with previous data for other risperidone formulations [40]. Of note, a high proportion of patients within this study had comorbid substance abuse (34%), strengthening the representativeness of these findings for understanding the real-world tolerability of risperidone ISM^®^ in this population [40].

As with other D_2_ receptor antagonists, risperidone can increase prolactin levels, which can have adverse effects, including sexual and reproductive dysfunction and a risk of fractures [38,44,45]. In both the main and open-label phases of the PRISMA-3 study, and from real-world experience from the RESHAPE study, prolactin-related TRAEs were observed to be infrequent in patients receiving risperidone ISM^®^ [38,39,40]. However, these findings should be interpreted with caution. As is the standard procedure for clinical studies, both the PRISMA-3 study and the real-world RESHAPE study relied on self- or investigator-reported potential prolactin-related symptoms, such as headache, decreased libido, or oligomenorrhea [38,40]. Thus, clinically relevant prolactin-related symptoms may have been under-recognized, and so the reported rates of prolactin-related adverse events may not fully reflect the prevalence of biochemical hyperprolactinemia or associated clinical manifestations.

Additional insights come from a Spanish retrospective, cross-sectional, real-world study, where clinically significant hyperprolactinemia was observed in 56% of patients treated with risperidone ISM^®^ and 69% of patients treated with monthly paliperidone palmitate, with no significant difference between treatment type [46]. Sexual dysfunction rates were also similar between risperidone ISM^®^ and paliperidone palmitate (63% versus 72%, *p* = 0.465); however, women receiving paliperidone palmitate showed higher prolactin levels than those treated with risperidone ISM^®^ [46].

Further investigation is warranted to better characterize the relationship between prolactin elevations, associated symptoms, and their clinical relevance, ideally utilizing rating scales for sexual dysfunction [47].

## 3. Bridging the Gap: From PK/PD to Clinical Practice

### 3.1. Initiation and Early-Phase Management

Although several clinical studies have described the PK/PD profile of risperidone ISM^®^ [31,32,33], there remains limited awareness of these data and how to translate them into clinical decision-making.

In the Phase 1 multicenter, open-label, randomized PRISMA-1 trial, in which patients with schizophrenia were treated with up to 100 mg of risperidone ISM^®^, mean plasma concentrations of the active moiety were 29.6 ng/mL at 24 h and 31.4 ng/mL at 48 h, showing rapid attainment of therapeutic plasma levels without oral supplementation or a booster shot [33]. These initial findings were substantiated by the Phase 2 PRISMA-2 study, in patients with schizophrenia who received multiple doses of risperidone ISM^®^ 75 mg in both gluteal and deltoid muscles. Therapeutic concentrations of the active moiety were achieved within 2 h of administration and peak concentrations were attained between 24 and 48 h [31]. Peak concentrations ranged from 39.6 to 53.2 ng/mL after gluteal administration and 54.1–61 ng/mL after deltoid administration [31]. In another Phase 1 study (BORIS-1), participants first received 1 week of oral risperidone 4 mg, followed by four monthly doses of risperidone ISM^®^ 100 mg IM [32]. Results showed that 12 h after the first injection, mean active moiety plasma concentrations achieved levels similar to those observed at steady state with oral risperidone treatment. The BORIS-1 study also showed that steady-state concentrations of risperidone ISM^®^ were achieved following the first dose [32].

Although rapid attainment of therapeutic levels of risperidone ISM^®^ has been demonstrated, this should not be interpreted as implying immediate clinical response. In the Phase 3 PRISMA-3 study, risperidone ISM^®^ 75 mg and 100 mg resulted in statistically significant efficacy versus placebo as early as Day 8 after the first injection and is generally well tolerated [38]. However, no statistically significant advantage in efficacy responders was observed at Day 4 (the first assessed time-point), despite early attainment of therapeutic plasma concentrations [38]. Clinically, this distinction is important: although PK exposure is achieved within hours, measurable symptomatic improvement depends on downstream PD, clinical and behavioral changes that may emerge only after several days [48]. Therefore, the early PK profile of risperidone ISM^®^ should be understood as providing rapid antipsychotic coverage without the need for oral supplementation, rather than guaranteeing immediate symptom resolution. This is particularly relevant during treatment initiation, hospital discharge planning, and transitions from oral therapy, where clinicians need confidence that PK exposure is present while continuing to monitor clinical response over the following days and weeks. Notably, the delayed clinical action compared with the PK properties applies to all medications for psychosis and is not specific to risperidone ISM^®^ [48].

The results from the risperidone ISM^®^ PK clinical program are further supported by real-world clinical evidence, although the observational nature of this study means that any interpretation should consider potential biases and confounders. This study observed that therapeutic levels of risperidone were reached within 2 h post-injection, with median Tmax occurring at 24 h, and Cmax values of 55.7 ng/mL for 75 mg and 59 ng/mL for 100 mg across gluteal and deltoid injection sites [49].

Together, these studies reported that risperidone ISM^®^ can be administered in either the gluteal or deltoid muscle without oral supplementation or a loading dose, while rapidly achieving therapeutic exposure and steady-state concentrations [31,32]. This may simplify use in the early phases of treatment, including in inpatient settings, at discharge, and during transitions from oral medications for psychosis, while facilitating earlier assessment of tolerability and subsequent dose optimization.

#### Expert Recommendations

- Risperidone ISM^®^ can be initiated without oral supplementation or loading doses in patients for whom risperidone treatment is appropriate, and tolerability has been established [32]. This may simplify treatment initiation, reduce regimen complexity, and support continuity of care, particularly during emergency room visits, hospitalization, discharge planning, or transition from oral risperidone [32].
- Therapeutic plasma concentrations are reached within hours of injection, with significant efficacy observed as early as Day 8, enabling early assessment of tolerability and clinical response [31,32,33,38].
- The injection can be given into either the gluteal or deltoid muscles, delivering comparable plasma concentrations [31].
- Steady state is achieved after the first dose, allowing for greater simplicity and agility in dose adjustments [32].

### 3.2. Dose Conversion and Switching Strategies

Switching treatments is common in schizophrenia management [50], despite complex layers to consider for receptor and PK profiles, dose equivalencies, dose conversions, and switching between oral medications for psychosis and LAIs [50,51]. As switching between medications for psychosis with different PD and PK properties may increase the likelihood of rebound symptoms or withdrawal effects, evidence-, PD- and PK-based antipsychotic switching strategies should be considered for all patients changing medications [50,51].

Determining dosing equivalencies between oral and LAI medications for psychosis or between LAIs is often difficult because most of them have long half-lives, making traditional bioequivalence studies time-consuming [52]. Some clinical dose equivalencies for LAIs have been reported in the Second International Consensus Study of Antipsychotic Dosing [53]. There is also evidence that population PK modeling may be used to demonstrate bioequivalences, showing promise as an option for determining dose conversions with LAIs, although this may be complicated by different delivery technologies such as ISM^®^ [52].

Understanding the PK and PD properties of medications for psychosis can help inform switch strategies [21,25,50]. For example, switching between medications with comparable PK and PD properties can be done relatively easily by cross-titration. However, switching between pharmacologically distinct molecules should account for differences in half-lives, receptor-binding profiles, as well as PD (e.g., antagonism versus partial agonism) to avoid symptom worsening or adverse events [50].

**Figure 2:**
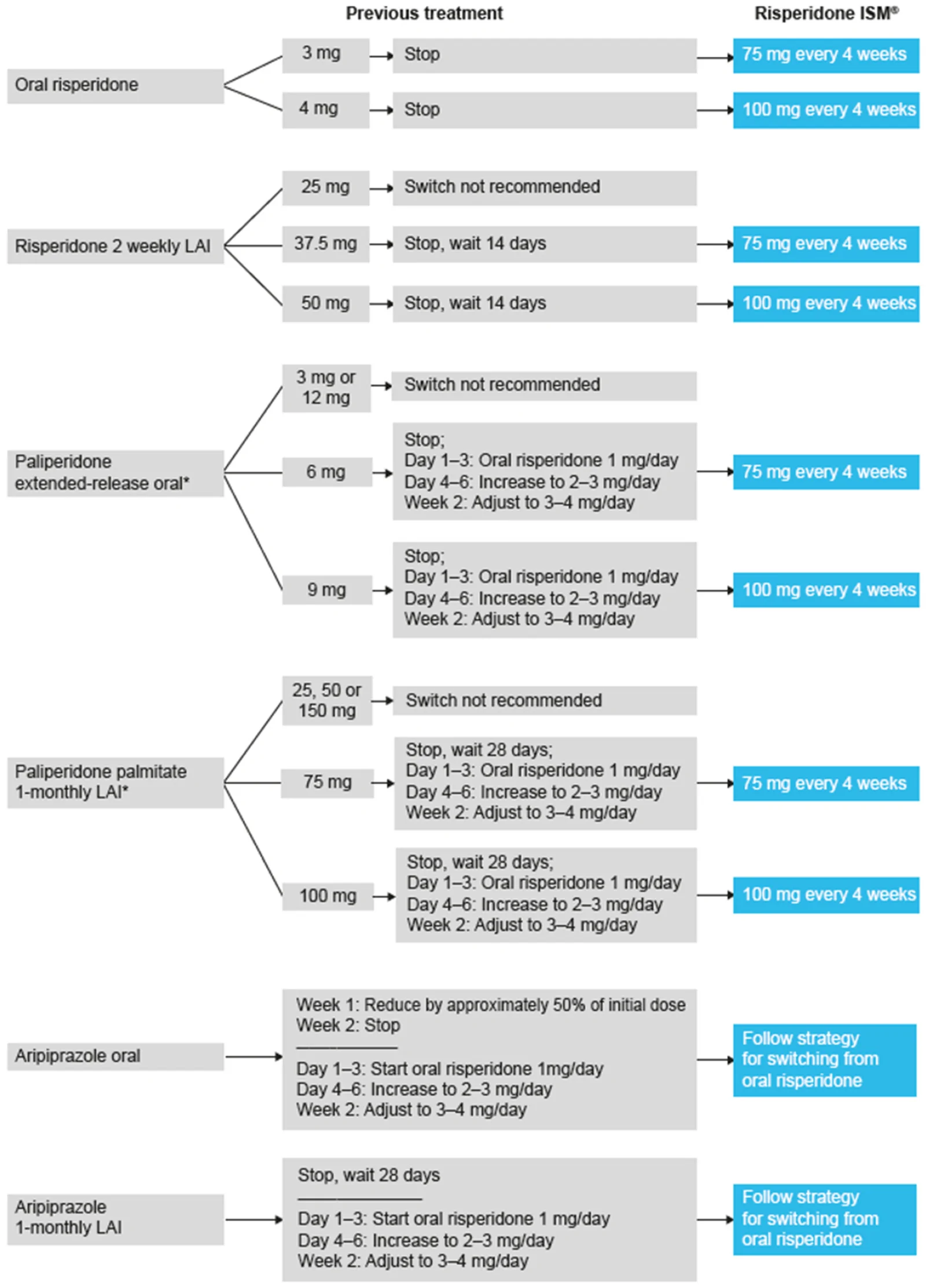
Schematic overview of dose conversion and switching strategies for risperidone ISM^®^ [54]. SwitchRx can be used to support individualized treatment planning when switching from oral medications for psychosis or other LAI formulations to risperidone ISM^®^. Prior tolerability to risperidone should be established before risperidone ISM^®^ initiation in patients without previous exposure. * For paliperidone palmitate, some protocols do not include the oral tolerability test (e.g., SwitchRx [54]); physicians should consider local recommendations and individual cases when switching. LAI, long-acting injectable.

Online tools, such as SwitchRx, can be useful for determining switching strategies for medications for psychosis, and for many other medications [54]. Below, we have summarized how SwitchRx can be used to evaluate a treatment plan for a patient switching from various formulations to risperidone ISM^®^ (Figure 2) [54].

As with all LAI medications for psychosis, risperidone ISM^®^ cannot be removed after administration, and drug exposure may persist after treatment is discontinued. Tolerability to oral risperidone should therefore be established before initiation in patients without prior exposure [30]. Suspected neuroleptic malignant syndrome (NMS), severe EPS, or serious hypersensitivity requires immediate assessment, discontinuation or withholding of further antipsychotic exposure, and supportive care, dependent on syndrome, severity, contraindications, and even local emergency protocols [55]. Available evidence has not indicated worse NMS outcomes with LAI than with oral medications for psychosis in terms of mortality [55,56,57], although prompt recognition and treatment remain essential.

Both the European Summary of Product Characteristics and Canadian Product Monograph agree that the approximate equivalence for an oral risperidone dose of 3 mg/day is risperidone ISM^®^ 75 mg. For oral risperidone ≥ 4 mg, the European guidance states that risperidone ISM^®^ 100 mg is the equivalent dose, supported by the observation that ≥65% D2 receptor occupancy is reached at this point, indicating that it has achieved the threshold for therapeutic efficacy [35,58,59]. In this context, it may be more appropriate to discuss dose ranges rather than exact dosing equivalences. However, it is also important to note that the Canadian Product Monograph diverges from the European guidance, stating that risperidone ISM^®^ 100 mg is equal to oral risperidone 4 mg only, showing a localization contrast in dose guidance. Furthermore, risperidone ISM^®^ may not be appropriate for patients stabilized on low doses of oral risperidone (<3 mg), although this requires further investigation [58,59].

If a patient is not responding to risperidone ISM^®^ initially, “pseudo-resistance” should be considered, particularly when rapid metabolism, anatomical- or administration-related factors may prevent attainment of therapeutic concentrations. Assessment should also include adequate dosing, prior adherence to oral medication, comorbidities, and diagnosis reassessment [60,61].

Routine therapeutic drug monitoring (TDM) of risperidone ISM^®^, while potentially optimal, may not be feasible in many usual-care settings [62,63]. There is currently insufficient evidence to formulate concentration-based risperidone ISM^®^ dose-adjustment algorithms or to recommend routine off-label interval shortening (particularly considering the biphasic PK release pattern) or oral supplementation. In addition, clinical studies and real-world evidence of risperidone ISM^®^ indicate a favorable tolerability and safety profile, with low risk of relapse, potentially indicating that heterogeneity in serum level fluctuations does not lead to clinical undue events [31,38,39,40].

However, TDM may be considered in selected cases, including unexpectedly poor response, suspected excessive exposure or adverse effects, possible drug–drug interactions, uncertainty regarding administration, or relevant renal or hepatic impairment [62]. Results should be interpreted according to the timing of sampling relative to the injection and using formulation-specific PK information [62]. Any management decision should integrate symptoms, adherence and administration history, sampling time, interacting medications, organ function, and product-label recommendation.

#### Expert Recommendations

- Clear guidance on dose conversions, timing, and switching schemes is essential to support informed treatment decisions and mitigate adverse events. Switching to risperidone ISM^®^ should be guided by previous medication exposure, assessment of “pseudo-resistance”, prior tolerability, current clinical stability, relapse risk, and the urgency of ensuring treatment continuity. Tools such as SwitchRx can be useful to develop effective switching strategies.
- For patients already stabilized on oral risperidone, risperidone ISM^®^ can be initiated directly, without oral supplementation or loading dose. A dose of 75 mg every 4 weeks corresponds approximately to oral risperidone 3 mg/day, while 100 mg every 4 weeks corresponds approximately to oral risperidone ≥ 4 mg/day [59,64].
- For patients with no prior exposure to risperidone or paliperidone, a short oral risperidone trial should generally be considered before risperidone ISM^®^ initiation to at least confirm tolerability. This period should primarily be interpreted as a tolerability assessment rather than a definitive test of efficacy.
- For patients switching from another risperidone LAI, the timing of risperidone ISM^®^ initiation should account for the previous formulation’s dosing interval and residual exposure. The next risperidone ISM^®^ dose should generally be aligned with the time the next LAI dose would have been due, while avoiding unnecessary overlap or prolonged gaps in medication coverage.
- For patients switching from non-risperidone or non-paliperidone medications, the strategy should account for differences in receptor profile, half-life, as well as prior history of tolerability to minimize either duplicative side effects when overlapping with medications with similar PD profiles for too long, or to minimize rebound phenomena when discontinuing the medication with distinct receptor blocking PD profiles too fast [25]. One strategy is to begin with a relatively short oral risperidone phase before commencing risperidone ISM^®^ to facilitate the switching process with more dose-adjustment opportunities, particularly when switching from medications with distinct PD profiles, such as partial dopamine agonists or agents with strong antihistaminergic, anticholinergic, or antiadrenergic effects.
- During and after the switch, clinicians should monitor for relapses, withdrawal or rebound symptoms, early adverse effects, and signs of inadequate exposure. Apparent lack of response should prompt assessment of dose adequacy, injection timing, adherence history, comorbidities, and diagnostic factors, before concluding treatment resistance.

### 3.3. Time to and Within Therapeutic Concentrations

A potential barrier to the use of risperidone ISM^®^ is understanding how therapeutic levels are maintained, and whether receptor occupancy is sustained over the full 4-week dosing interval, particularly in relation to concerns about breakthrough symptoms towards the end of the interval reported with other LAIs [65].

Clinical evidence shows that the Tmax for risperidone ISM^®^ 100 mg at steady state occurs at a median of 48 h, with steady-state concentrations of 21.22–64.85 ng/mL, consistent with concentrations observed following oral risperidone treatment 4 mg/day (19.39–54.08 ng/mL) [32]. Importantly, achieving these steady-state concentrations results in sustained active moiety plasma concentrations within the therapeutic window throughout the 28-day dosing interval, without a clinically meaningful decline towards the end of the interval [32]. This sustained exposure is supported by findings from the pivotal 12-week PRISMA-3 study in patients with an active exacerbation or relapse of schizophrenia, in which statistically significant improvements versus placebo were observed as early as Day 8 with the 100 mg dose and Day 15 with the 75 mg dose. These differences were maintained to the end of the study [38]. Collectively, these findings support sustained therapeutic exposure and antipsychotic efficacy throughout the full dosing period.

A study using PK/PD modeling reported that, at steady state, median D2 receptor occupancy for risperidone ISM^®^ 75 mg and 100 mg remained above 65% throughout the 28-day dosing interval and showed lower variability than oral risperidone [66]. Furthermore, D2 receptor occupancy within the therapeutic range of 65–80% was reached within a few hours after injection, and modeling revealed symptomatic improvement over time following drug administration [66]. Another PK modeling study also showed that, during the 28-day dosing period, the median time below 65% D2 receptor occupancy was 0 days for risperidone ISM^®^ [35]. In the same study, modeling of paliperidone palmitate showed that at the end of the first monthly injection of 100 mg at Week 5, 50% of patients were below the 65% D2 receptor occupancy threshold. Together, these data suggest that D2 receptor occupancy is maintained above 65% throughout the dosing interval with risperidone ISM^®^, supporting sustained antipsychotic coverage and reducing the theoretical risk of end-of-dose breakthrough symptoms.

Another factor to consider when evaluating risperidone ISM^®^ exposure across the dosing interval is the presence of “peak-to-trough” fluctuations, which may influence antipsychotic tolerability. An increase in the plasma concentration peak-to-trough ratio, calculated as a ratio of mean maximum (Cmax) to mean minimum (Cmin) plasma concentration at steady state, may result in D2 receptor occupancy fluctuations and affect tolerability [67]. Using the values from the PK study published in Walling et al., oral risperidone 4 mg is estimated to have a peak-to-trough ratio of 2.79, similar to that of risperidone ISM^®^ 100 mg (3.06) [32]. By contrast, the PRISMA-2 study reported a ratio of 3.76 for gluteal administration of risperidone ISM^®^ 75 mg [31]. Given the variation seen across studies, further investigation of potential fluctuations at both 75 mg and 100 mg doses is needed to determine their clinical relevance and potential effect on D2 receptor occupancy.

It should be noted that 1 week after the end of the recommended dosing interval (4 weeks), plasma concentrations of risperidone ISM^®^ have been shown to decline by approximately 50% [58]. Therefore, the Canadian Product Monograph specifies that to avoid missing the 4-week dose interval, patients may be given the injection up to 3 days before the dosing interval. However, if a dose is missed, risperidone ISM^®^ can be restarted immediately without overlap or a booster injection, facilitating treatment reinitiation and continuity of care.

#### Expert Recommendations

- Therapeutic D2 receptor occupancy is expected to be achieved within hours of risperidone ISM^®^ administration and maintained throughout the 28-day dosing interval, supporting sustained symptom control and potentially reducing the risk of end-of-dose breakthrough symptoms [35].
- While therapeutic exposure is maintained across the approved dosing interval, plasma concentrations may decline substantially if the next injection is delayed by 1 week or more. Injections should therefore be scheduled every 4 weeks, with consideration of administration up to 3 days before the planned date when clinically or logistically needed.
- If a dose is missed, risperidone ISM^®^ can be restarted without oral supplementation or a booster dose, which may simplify reinitiation and reduce treatment gaps.
- Patients reporting breakthrough symptoms toward the end of the dosing interval should be carefully assessed for injection timing, missed or delayed doses, symptom trajectory, comorbid substance use, psychosocial stressors, and relapse risk before assuming inadequate drug exposure.
- Routine shortening of the dosing interval is not generally supported by the current evidence; however, dosing intervals should be clinically reviewed in patients with recurrent end-of-dose symptoms, repeated delays, or concerns about tolerability or exposure.

### 3.4. Adequacy of Exposure at High and Low Oral Equivalent Dose of Risperidone

When considering a switch to risperidone ISM^®^, some patients may be stabilized on oral risperidone doses higher than 4 mg/day or lower than 3 mg/day. In these situations, it is important to determine whether available risperidone ISM^®^ doses are likely to provide adequate coverage while avoiding excessive exposure.

For patients receiving higher doses of oral risperidone (i.e., ≥4 mg), PK modeling of risperidone ISM^®^ 100 mg shows that D2 receptor occupancy is maintained above 65% throughout the dosing period, which is consistent with perfect adherence to oral risperidone at 4–8 mg/day. However, the time below this 65% threshold increases with intermediate to poor adherence to oral risperidone 4–8 mg/day, highlighting the advantage of an LAI in maintaining receptor occupancy over time [35]. Additionally, in the extension of the PRISMA-3 trial, 99 patients previously treated with ≥4 to ≤6 mg/day of oral risperidone were switched to risperidone ISM^®^ 100 mg, and symptomatic improvement was observed across patients [39]. TRAEs in this subgroup were uncommon and included akathisia (4%), gynecomastia (1%), hepatic steatosis (1%), hepatocellular injury (1%), hyperprolactinemia (1%), and increased body weight (3%) [39]. Together, these data suggest risperidone ISM^®^ 100 mg can provide exposure and D2 receptor occupancy comparable to higher doses of oral risperidone, with less fluctuation over time [35].

For patients receiving low doses of oral risperidone (<3 mg/day), evidence is more limited. Current product recommendations support switching patients stabilized on 3 mg/day or higher with risperidone ISM^®^ 75 mg, corresponding to approximately oral risperidone 3 mg/day. Therefore, in patients who are stable and well controlled on <3 mg/day, switching to risperidone ISM^®^ may result in higher exposure than their current oral regimen. Although the overall safety and tolerability profile of risperidone ISM^®^ is generally consistent with known risperidone effects [31,38,40], the decision to switch these patients should be individualized, considering relapse history, adherence concerns, prior tolerability, sensitivity to dose-related adverse effects, age, renal function, and patient preference. In patients with substantial adherence concerns or repeated relapses despite apparent low-dose treatment, risperidone ISM^®^ may still be considered, but clinicians should monitor carefully for dose-related adverse effects, including extrapyramidal symptoms, sedation, prolactin-related effects, and orthostatic hypotension [43]. Further clinical evidence is needed to better define the role of risperidone ISM^®^ in patients stabilized on low doses of oral risperidone below 3 mg/day.

#### Expert Recommendation

- Patients stabilized on oral risperidone ≥ 4 mg/day can generally be switched to risperidone ISM^®^ 100 mg every 4 weeks, with evidence supporting maintenance of therapeutic D2 receptor occupancy and potential improvement in symptom stability [35,39].
- For patients receiving oral risperidone 3 mg/day, risperidone ISM^®^ 75 mg every 4 weeks represents the recommended approximate equivalent dose.
- For patients stabilized on oral risperidone < 3 mg/day, switching to risperidone ISM^®^ is not indicated and should not be considered routine. It may be reasonable in select cases with shared decision-making and informed consent where adherence concerns, relapse risk, or patient preference favor an LAI, but the potential for higher exposure and dose-related adverse effects from this off-label use should be discussed and monitored.

### 3.5. Comparative Understanding Versus Other LAIs

Data from the risperidone ISM^®^ clinical program show that it may be an effective and well-tolerated treatment for schizophrenia, including in patients previously treated with oral risperidone [32,39]. However, direct comparisons to other LAI medications have been limited due to the relatively recent approval of risperidone ISM^®^; large comparative studies will require several years of widespread use for meaningful comparisons to be made [66].

The basic characteristics of selected second-generation LAIs are shown in Table 1. Current evidence suggests that there is not a large significant advantage between different LAIs in terms of symptom improvement and relapse rate [26]; however, there are significant variations in adverse event profiles (Table 2) [43], practical implications such as frequency of injection and reconstitution procedures (Table 1), drug–drug interactions, as well as in initiation and switching strategies [2,25]. Selecting the most appropriate LAI medication depends on balancing differences in pharmacology, tolerability, administration, and patient-centered factors to optimize patient adherence and outcomes [68,69]. Differences in initiation and switching strategies among LAI medications are driven by their distinct pharmacokinetic and pharmacodynamic properties, with some drugs requiring oral supplementation or loading doses to achieve therapeutic concentrations while others can be initiated or switched using simplified regimens (Table 1) [25]. 

In terms of adverse events, side effects correlate with their mechanism and receptor-binding profile. Risperidone and paliperidone are associated particularly with prolactin elevation and dose-related EPS; aripiprazole formulations may have lower prolactin liability but greater vulnerability to akathisia in some patients; and olanzapine formulations may carry greater sedation and weight-gain liability (Table 2) [43]. However, evidence directly comparing risperidone ISM^®^ with these LAIs remains limited.

In one systematic review comparing risperidone ISM^®^ with other once-monthly LAI formulations, including aripiprazole monohydrate and paliperidone palmitate, participants treated with risperidone ISM^®^ had a significantly lower incidence of extrapyramidal symptoms (EPS) than those treated with aripiprazole monohydrate (OR: 0.25 [95% CI 0.12–0.53], *p* < 0.001). Use of anticholinergic agents (an indirect measure of EPS) to treat these symptoms was also reported to be significantly lower with risperidone ISM^®^ compared with both aripiprazole monohydrate (OR: 0.01 [95% CI 0.003–0.06], *p* < 0.001) and paliperidone palmitate (OR: 0.29, [95% CI 0.10–0.83], *p* = 0.021) in a matching-adjusted but only indirect analysis that may be imprecise [66]. In another meta-analysis, risperidone and paliperidone LAI formulations were both found to be associated with less use of antiparkinsonian medication than their oral counterparts [70]. However, results from a large, recently published network meta-analysis show risperidone (primarily oral, but including some LAI formulations) was associated with an OR of 1.89 for use of antiparkinsonian medication versus placebo and showed no pooled difference to aripiprazole in terms of akathisia. It should be noted here that while akathisia is often included within the range of extrapyramidal symptoms, it does not respond well to anticholinergics and thus forms a separate measure [71]. Data from clinical trials have shown that drug-induced akathisia varies significantly by medication, with risperidone showing a comparatively low risk compared to others [72]; however, a large meta-analysis showed that risperidone was one of 11 medications, including aripiprazole and haloperidol, that displayed higher odds of akathisia compared to at least three other medications for psychosis [42]. Conversely, another network meta-analysis demonstrated no significant difference between oral and LAI forms of risperidone compared to placebo with regard to akathisia (risperidone LAI risk ratio: 0.97 [95% CI 0.49, 1.92]; risperidone-oral risk ratio: 1.26 [95% CI 0.67, 2.38] and no significant difference in risk ratio in a head-to-head comparison [73]. In the same network meta-analysis, it was found that both forms of risperidone showed significantly similar risk ratios for akathisia in comparison to other medications for psychosis, including aripiprazole, with both potentially associated with a lower risk than haloperidol [73]. This lack of consensus among studies suggests that further investigation is needed to determine the risk of EPS and akathisia with risperidone ISM^®^ versus placebo and versus oral risperidone. In a modeling study, paliperidone palmitate was predicted to have a slower rise in D2 receptor occupancy than risperidone ISM^®^, with a consistently lower proportion of patients achieving >65% D2 receptor occupancy across consecutive injections [35]. Since a 65% D2 receptor occupancy is a well-established biomarker corresponding to the lower threshold generally associated with clinical antipsychotic efficacy [35], such difference may translate clinically.

While no head-to-head trials have directly compared risperidone ISM^®^ with paliperidone palmitate, a network meta-analysis comparing both oral and LAI forms of second-generation medications for psychosis found that risperidone LAI was more efficacious than paliperidone LAI in improving schizophrenia symptoms and, along with oral olanzapine, more efficacious than oral aripiprazole [70]. Similarly, a large meta-analysis comparing the efficacy and tolerability of 19 second-generation medications for psychosis in acute schizophrenia demonstrated that risperidone (including both oral and LAI formulations) was among the four medications, along with clozapine, amisulpride and olanzapine, to have greater efficacy for overall symptom reduction than at least three others [42]. Additionally, a recent randomized trial comparing seven medications for psychosisin acute schizophrenia found that olanzapine and risperidone had the largest effects on positive and negative symptoms at 6 weeks compared with aripiprazole, ziprasidone, and quetiapine [74].

Another element to consider is comparisons to equivalent oral formulations of medications for psychosis. Evidence from a nationwide study has shown that, at optimal dose ranges, several LAI medications, including risperidone, provided greater protection against relapse than their equivalent oral formulations [75]. A meta-analysis of 66 studies also concluded that LAI medicationswere generally as effective as their oral formulations, with a potential for fewer side effects, but the indirect nature of this study precludes firm conclusions. Interestingly, in this study, risperidone LAI, subcutaneous risperidone LAI, and risperidone ISM^®^ showed similar adverse event outcomes as each other [70].

Together, these data suggest that risperidone ISM^®^ has a predictable PK/PD profile, rapidly reaches therapeutic levels, maintains D2 receptor occupancy across the dosing interval, and is associated with meaningful symptomatic improvement and a tolerability profile consistent with previous risperidone formulations. However, given the limited number of direct head-to-head comparisons with other LAIs, treatment selection should remain individualized and consider prior response, tolerability, patient preference, dosing logistics, and the need for rapid therapeutic exposure.

#### Expert Recommendations

- LAI selection should consider factors beyond dosing interval and time to therapeutic exposure. Relevant factors include evidence for acute and maintenance treatment, receptor-binding profiles, administration characteristics, practical considerations (e.g., reconstitution, storage, oral supplementation/loading strategies and post-injection observation), clinically relevant drug–drug interactions and procedures for delayed or missed doses. No individual LAI is preferable across all dimensions.
- Risperidone ISM^®^ should be considered alongside other LAIs for the treatment of schizophrenia, particularly when rapid attainment of therapeutic exposure, simple initiation without oral supplementation or loading doses, and sustained coverage across the dosing interval are clinical priorities.
- When selecting LAIs, clinicians should consider not only efficacy but also initiation requirements, time to therapeutic exposure, time to steady state, injection interval, need for oral overlap or booster injections, post-injection monitoring requirements, prior treatment response, tolerability history and patient preference.
- Risperidone ISM^®^ may be particularly useful in clinical situations where treatment continuity needs to be established quickly, such as after hospitalization, during transition from oral risperidone, or when oral adherence is uncertain.
- The available comparative evidence suggests a favorable tolerability profile for risperidone ISM®, although direct head-to-head evidence remains limited.
- Ultimately, LAI choice should be individualized through shared decision-making, balancing pharmacological properties, clinical goals, adverse-effect vulnerabilities, administration logistics, and patient preferences.

## 4. Addressing Remaining Uncertainties

Although clinical and real-world evidence may support the use of risperidone ISM^®^ for the treatment of schizophrenia [31,33,40,49], some uncertainties remain regarding interindividual variability, effectiveness in specific populations, and longer-term safety.

When initiating risperidone ISM^®^ in elderly patients, a 75 mg starting dose should be considered if deemed clinically appropriate. For other vulnerable populations, such as patients with moderate to severe renal impairment, hepatic impairment, or in individuals under 18 years of age, risperidone ISM^®^ has not yet been systematically studied [64]. Patients with impaired renal and hepatic function may have reduced ability to eliminate the active antipsychotic fraction, resulting in increased plasma concentrations and a potentially higher risk of adverse events. In these patients, careful titration with oral risperidone before initiating treatment with risperidone ISM^®^ 75 mg may be considered in specific cases [54,59]. The European Summary of Product Characteristics states that for patients with mild renal impairment (creatinine clearance 60–89 mL/min), no dose adjustment of risperidone ISM^®^ is required, but the product is not recommended in patients with moderate to severe renal impairment (creatinine clearance < 60 mL/min).

Regarding interindividual variability, one real-world PK study of risperidone ISM^®^ reported high variability in mean plasma concentrations, with 45.4% of patients exhibiting trough levels outside the therapeutic range [49]. This variability may hypothetically reflect differences in reconstitution processes or interindividual differences in body fluid composition at the injection site, potentially leading to different rates of polymer degradation [49]; however, more evidence is needed to draw any firm conclusions. There is also evidence that sex and body mass index (BMI) may influence risperidone ISM^®^ PK, with females and patients with higher BMI having lower clearance values and thus higher risperidone plasma levels, suggesting that some patients may require lower dosages. However, sex and BMI did not affect total symptom score in the PRISMA-3 trial; therefore, the clinical significance of reduced clearance remains uncertain and requires further study [34]. Because correct reconstitution and intramuscular administration are important for reliable delivery, healthcare professionals should receive formulation-specific training and follow the manufacturer’s preparation and injection instructions. When exposure or response is unexpected, reconstitution procedure, injection technique, injection site, needle selection, dosing interval, and delayed or missed administrations should be reviewed.

Established patient-related risk factors should also be considered when assessing the likelihood of adverse events to risperidone ISM^®^. For parkinsonism symptoms, these include higher cumulative dose or exposure, older age, female sex, non-European ancestry, dementia, hypertension, and pre-existing parkinsonism. Clinically significant hyperprolactinemia is more likely in younger female patients, while weight gain is more common in individuals with baseline increased BMI, female sex, younger age, and increased value of parents’ BMI. Importantly, these risk factors are associated with medications for psychosisin general and should not be interpreted as conferring risks specific to risperidone ISM^®^.

As risperidone ISM^®^ is more recently approved and less widely available than some other LAIs, long-term data remain relatively limited. However, risperidone ISM^®^ remained effective with a favorable safety profile up to 1 year follow-up in the PRISMA-3 extension study [39]. In addition, oral risperidone has been widely used for several decades, supporting confidence in the broader long-term safety and effectiveness of risperidone as a therapeutic agent [76,77]. Nevertheless, continued pharmacovigilance and real-world studies are needed to clarify longer-term outcomes specifically with the ISM^®^ formulation.

From a clinical perspective, these uncertainties should not prevent appropriate use of risperidone ISM^®^, but they should guide individualized prescribing. Caution may be warranted, indeed for all medications for psychosis, in patients with renal or hepatic impairment, older adults with frailty or polypharmacy, patients with prior dose-related adverse effects, and those in whom higher exposure may be clinically relevant. In these cases, clinicians should consider oral tolerability testing, more frequent follow-up during the early treatment period, and systematic monitoring for EPS, prolactin-related adverse effects, sedation, orthostatic hypotension, and metabolic changes.

## 5. Future Directions

Future research on risperidone ISM^®^ should prioritize clinically relevant questions that can directly inform treatment selection and day-to-day prescribing. These include expanding real-world evidence, conducting direct comparative studies with other LAIs or oral medications for psychosis, and evaluating comprehensive switching strategies from oral medications for psychosis, paliperidone formulations, and other LAIs to risperidone ISM^®^.

Further studies are also needed to clarify how risperidone ISM^®^ performs in patient groups commonly encountered in clinical practice but underrepresented in trials, including older adults, ethnically diverse populations, patients with renal or hepatic impairment, people receiving lower oral risperidone doses, young patients with early-stage illness, and those with complex psychiatric comorbidities, including substance-use disorder.

Developing more personalized dosing strategies to tailor treatment to individual patient needs and clinical profiles will also be essential. Future work should examine whether clinical characteristics such as prior symptomatic response, adverse-effect vulnerability, renal function, BMI, sex, adherence history, symptom severity, and patient preference can help guide dose selection, level of monitoring, and switching approaches. Pragmatic studies embedded in routine care may be particularly useful to determine how the PK/PD advantages of risperidone ISM^®^ translate into outcomes that matter to patients, including relapse prevention, functioning, treatment satisfaction, quality of life, and continuity of care.

## 6. Conclusions

Risperidone ISM^®^ offers a PK/PD profile that, when properly understood and applied, could support simplified initiation, switching, and dose optimization, and confidence in sustained therapeutic exposure across the dosing interval. By reaching therapeutic concentrations rapidly without oral supplementation or loading doses, while maintaining D2 receptor occupancy over 4 weeks, risperidone ISM^®^ may be able to address several practical barriers that have limited LAI use in clinical practice.

In this article, we have provided actionable recommendations from experts in the field based on the available evidence and their clinical experience. However, these should not be interpreted as current treatment guidelines from governing bodies. For clinicians, the main value of risperidone ISM^®^ lies not only in risperidone’s efficacy and PK/PD profile, but in how this profile can be translated into practical decisions: initiating treatment more simply, supporting continuity of care after relapse or hospitalization, reducing uncertainty during switching, and helping patients maintain symptom stability over time. Used within an individualized, patient-centered treatment plan, risperidone ISM^®^ represents an important LAI option for the ongoing management of schizophrenia.

## Figures and Tables

**Table 1:** Comparative profile of selected LAI medications for psychosis for the treatment of schizophrenia with injection intervals up to every month [2,23,24].

	Aripiprazole Monohydrate (Once Monthly) *^‡^	Olanzapine Pamoate (Every 2 Weeks or Monthly) **^‡^	Paliperidone Palmitate (Once Monthly) ^†^	Risperidone (Every 2 Weeks) ^‡^	Risperidone Subcutaneous (Once Monthly) **^‡^	Risperidone ISM^®^ (Every 4 Weeks) ^‡^
**Oral** **supplementation or booster** **injection**	Yes, two options	No	Yes, booster injection	Yes, oral supplementation	No	No
**Dose interval**	Monthly (no sooner than 26 days after previous injection)	2 or 4 weeks	Monthly	2 weeks	Monthly	Monthly
**Initiation** **regimen**	Option 1: One 400 mg injection and 10–20 mg oral aripiprazole for 14 days Option 2: Two injections of 400 mg at 2 separate sites and single dose of 20 mg oral aripiprazole	210 mg/2 weeks 300 mg/2 weeks 405 mg/4 weeks	Canadian label (mg eg.): Dose of 150 mg on treatment Day 1 and 100 mg on Day 8 (one week later), both administered in the deltoid muscle US label (mg of salt form): 234 mg on Day 1 and 156 mg on Day 8 (deltoid)	One 25 mg injection, overlap with oral risperidone or current medication for 3 weeks	50, 75, 100 or 125 mg (subcutaneous injection)	75 or 100 mg (gluteal or deltoid)
**Maintenance dose**	400 mg (300–400 mg)	300 mg/2 weeks	Canadian label (mg eg.): 75 mg (25–150 mg) US label (mg of salt form): 117 mg (39–234 mg)	25–50 mg	50–125 mg	75 or 100 mg
**Time to peak**	5–7 days	4 days	13 days	30 days	8–14 days	48 h (to first peak)
**Time to** **steady state**	300 mg: 3–4 months 400 mg: 4–8 months	3 months	8–9 months	8 weeks (after 4 injections)	6 months	4 weeks
**Storage** **requirements**	Room temperature (15–30 °C), protect from light	Room temperature (15–30 °C), protect from light	Room temperature (15–30 °C)	Refrigeration needed (2–8 °C) or room temperature for up to 7 days, protect from light	Refrigeration needed (2–8 °C) or room temperature for up to 90 days if unopened, protect from light	Room temperature (15–30 °C)
**Pharmacology**	Dopamine, serotonin	Dopamine, serotonin	Dopamine, serotonin, noradrenaline	Dopamine, serotonin, noradrenaline	Dopamine, serotonin, noradrenaline	Dopamine, serotonin, noradrenaline
**Mode of action**	Receptor partial agonist (D2, 5-HT1A)	Receptor antagonist (D2, 5-HT2)	Receptor antagonist (D2, 5-HT2, NAα-2)	Receptor antagonist (D2, 5-HT2, NAα-2)	Receptor antagonist (D2, 5-HT2, NAα-2)	Receptor antagonist (D2, 5-HT2, NAα-2)

* Also available in a formulation for administration every 2 months; ** Not available in Canada; ^†^ Also available in a formulation for administration every 3 months; **^‡^** Regimen the same for both Canadian and US label. The nature and extent of these limitations differ among currently available LAIs [21,25]. NA: noradrenergic.

**Table 2:** Selected adverse-effect profiles of selected medications for psychosis, with comparative side-effect relevance between drugs [43].

Associated Receptor	Adverse Event	Aripiprazole	Olanzapine	Paliperidone	Risperidone
D2	Acute parkinsonism	1	0–1	2	2
D2, 5-HT	Akathisia	2	1	1	2
D2	Tardive dyskinesia	0–1	0–1	0–1	0–1
D2	Hyperprolactinemia	0	1	3	3
D2, α1	Sexual dysfunction	0–1	2	2	2
D2, anticholinergic	Cognitive dysfunction	0	1	1	1
D2, 5-HT2C, H1	Weight gain	0	3	2	2
5-HT2C	Metabolic syndrome	0–1	3	1	1
H1	Sedation	0–1	1–2	0–1	1

Note: Numbers indicate comparative, not absolute, and side-effect relevance among drugs.

## Data Availability

No new data were created or analyzed in this study. Data sharing is not applicable to this article.

## Abbreviations

The following abbreviations are used in this manuscript:

BMI	Body mass index
CNS	Central nervous system
DMSO	Dimethyl sulfoxide
ECYM	Einstein Center for Youth Mental
EPS	Extrapyramidal symptoms
IM	Intramuscular
LAI	Long-acting injectable
OR	Odds ratio
PANSS	Positive and Negative Syndrome Scale
PD	Pharmacodynamic
PK	Pharmacokinetic
SmPC	Summary of Product Characteristics
TRAE	treatment-related adverse events
